# Association of Domain-Specific Physical Activity with Sleep Disorders in U.S. Adults: A Cross-Sectional Study Based on NHANES

**DOI:** 10.3390/healthcare14162476

**Published:** 2026-08-10

**Authors:** Rong Wang, Tangxiaoxue Zhang, Cailiang Zhou, Claudio L. Battaglini, Ting Jiang

**Affiliations:** 1 School of Sport Science, Beijing Sport University, Beijing 100084, China; 2020011945@bsu.edu.cn (R.W.);; 2 Key Laboratory of Sports and Physical Health Ministry of Education, Beijing Sport University, Beijing 100084, China; 3 Faculty of Psychology, Beijing Normal University, Beijing 100875, China; 4 Department of Exercise and Sport Science, University of North Carolina at Chapel Hill, Chapel Hill, NC 27599, USA

**Keywords:** domain-specific physical activity, sleep disorders, adult, NHANES

## Abstract

**Background:** The relationships of different physical activity domains [e.g., Occupation-related Physical Activity (OPA), Transportation-related Physical Activity (TPA), and Leisure-time Physical Activity (LTPA)] with sleep disorders (SD) remain poorly elucidated. This study investigated the associations of OPA, TPA, and LTPA with SD in American adults. **Methods:** Data were derived from 25,732 adults with a mean (standard deviation) age of 49.72 (17.53) years (12,692 male and 13,040 female) participating in the 2007–2018 cycles of the National Health and Nutrition Examination Survey (NHANES). The PA domains were assessed using the Global Physical Activity Questionnaire (GPAQ), and adherence to PA recommendations was determined according to the 2018 PA guidelines. SD were identified by self-reported or doctor-diagnosed questionnaire items. Survey-weighted logistic regression models were applied to evaluate the associations between PA domains and SD, and restricted cubic spline (RCS) analyses were conducted to examine potential nonlinear associations. **Results:** In the fully adjusted model, total PA was not significantly associated with SD (OR 1.01, 95% CI 0.89–1.13, *p* = 0.912). Regarding specific PA domains, TPA was associated with lower odds of SD (OR 0.79, 95% CI 0.67–0.93, *p* = 0.006), whereas OPA was associated with higher odds of SD (OR 1.15, 95% CI 1.02–1.30, *p* = 0.023). No significant association was observed between LTPA and SD (OR 0.96, 95% CI 0.85–1.09, *p* = 0.511). The multivariable-adjusted RCS analysis further indicated significant nonlinear associations between PA domains and SD (all *p* for non-linear <0.001). **Conclusion:** Our findings underscore a prominent “PA paradox” in sleep health among US adults, suggesting that active commuting (TPA) exerts a protective role against SD, whereas OPA serves as a potential behavioral risk factor. Future longitudinal studies and objective PA measurements are needed to validate these cross-sectional associations.

## 1. Introduction

Sleep is an essential physiological process that contributes to the maintenance of optimal physical functioning, emotional regulation, and overall health. Nevertheless, sleep disorders (SD) have become an increasingly recognized public health challenge worldwide. A substantial proportion of adults experience inadequate sleep duration or impaired sleep quality, which may result in considerable personal and societal consequences. In the United States, approximately 50–70 million individuals are affected by SD, highlighting the substantial burden of sleep-related problems [1]. Extensive evidence has demonstrated that poor sleep is associated with increased susceptibility to obesity [2], metabolic disorders [3], hypertension [4], and coronary heart disease [5]. Also, disturbed sleep patterns are closely related to adverse psychological outcomes, particularly depression and anxiety symptoms [6,7]. Beyond its health implications, inadequate sleep also generates considerable economic losses [8]. Given that sleep is influenced by multiple behavioral factors, such as physical activity (PA), diet, and smoking, increasing attention has been directed toward identifying modifiable lifestyle determinants of SD [9,10,11].

A robust body of literature consistently underscores the positive correlation between PA and optimal sleep outcomes in diverse populations [12,13,14]. Evidence from objective measurements has indicated that individuals with higher levels of PA tend to report better sleep quality. For example, an accelerometer-based investigation found that greater overall PA was associated with lower scores on the Pittsburgh Sleep Quality Index (PSQI), reinforcing a potential beneficial relationship between PA and sleep-related outcomes [12]. In another randomized controlled trial (RCT), children with attention deficit hyperactivity disorder (ADHD) who engaged in a 12-week jogging program (45 min/session, 1 session/week) exhibited improvements in sleep onset latency, sleep efficiency, and wake-after-sleep onset relative to a control group [13]. In addition, an updated umbrella review reported that the sleep-promoting benefits of PA extend throughout the entire lifespan and hold true for individuals with SD (16). Furthermore, in a study by Huang et al., the researchers concluded that increasing daily PA levels is beneficial for improving sleep in both healthy and comorbid populations with SD [15]. Collectively, existing evidence supports PA as a promising behavioral factor associated with sleep health.

Physical activity represents a multidimensional behavior encompassing various distinct domains [i.e., occupation-related PA (OPA), transportation-related PA (TPA), and leisure-time PA (LTPA)] [16]. Several studies have reported that the health consequences of PA may vary depending on the domains [17,18,19,20]. For instance, in a population-based study, it was reported that LTPA at any amount could contribute to a lower risk of depressive symptoms. However, OPA or TPA failed to exhibit comparable psychological benefits [17]. Similarly, Autenrieth et al. reported differential associations between domain-specific PA and mortality, showing that high levels of OPA, LTPA, and household PA were associated with reduced risks of cardiovascular disease mortality, while the protective role of TPA appeared less evident [19]. These discoveries suggest that the health-promoting effects of PA may differ substantially among domains. These divergent outcomes are partially elucidated by the “PA paradox” [21]. While LTPA is conducive to stress reduction and psychological relaxation, OPA typically demands repetitive movements and high psychological strain, which might lead to poorer health outcomes [22,23]. Additionally, TPA is predominantly characterized by active commuting via walking or bicycling and shifts activities outdoors. From the perspective of circadian gating, this outdoor exposure amplifies natural light reception, which plays a vital role in promoting sleep onset and improving sleep quality [24]. Hence, simply concentrating on LTPA or overall PA, which has been the focus of most previous research, may not be sufficient to elucidate the relationship between sleep and PA.

Some existing studies have explored the relationships between PA domains and various SD symptoms. Specifically, evidence from the Hispanic Community Health Study/Study of Latinos (HCHS/SOL) has revealed that moderate and high levels of TPA (e.g., walking or cycling) corresponded to a 16% and 36% reduction in the odds of mild obstructive sleep apnea (OSA), which is one of the most common SD symptoms. Likewise, compared to those who were inactive, participants who engaged in recreational moderate-to-vigorous PA (MVPA) had a lower risk of OSA [25]. On the contrary, a study conducted by Duan et al. observed no statistically significant deviations in OSA risk across three domains of PA (i.e., OPA, TPA, and LTPA) among Chinese adults [26]. In another study by Zheng and colleagues, based on a cohort of 0.5 million Chinese adults, the relationships between different PA domains and specific insomnia symptoms (e.g., daytime dysfunction and early morning awakening) varied significantly. The results of the study showed that while elevated OPA and LTPA mitigated insomnia risks in female participants, active commuting was unexpectedly tied to an increased likelihood of sleep disturbances [27]. These conflicting findings in previous articles may stem from study populations, measurement methods, and confounding adjustment strategies. In summary, previous studies regarding the associations between PA and SD remain inconclusive, and studies that identified the influence of domain-specific PA on SD, especially among American adults, are scarce. To address these inconsistencies, the present study distinguishes between occupational, transportation-related, and leisure-time PA and applies nonlinear dose–response modeling as well as subgroup analyses to better capture heterogeneous associations.

Furthermore, the associations of PA and SD are likely modulated by sociodemographic and biological characteristics. Prior investigations suggest that sleep characteristics vary across different age and sex strata, with females and elderly individuals reporting higher vulnerability to sleep disturbances [28,29]. Moreover, gender, age, body mass index (BMI), and alcohol consumption (for women only) exert significant influences on sleep-related outcomes [30]. Therefore, we hypothesize that the associations between domain-specific PA and SD may differ across several key subgroups.

This study sought to explore the associations between domain-specific PA (i.e., OPA, TPA, and LTPA) and SD among U.S. adults. Specifically, we aimed to (1) investigate how different domains of PA uniquely relate to SD; (2) examine potential dose–response associations between PA domains and SD; and (3) evaluate whether the relationship between the domain-specific PA and SD varied across sociodemographic and health-related subgroups, including age, gender, ethnicity, BMI, marital status, economic level, poverty income ratio, smoking status, and depression status.

## 2. Methods

### 2.1. Population and Data Sources

The present study was based on publicly available data from the National Health and Nutrition Examination Survey (NHANES). NHANES is an ongoing national survey designed to collect comprehensive information regarding health, nutrition, and lifestyle characteristics among the U.S. population. All NHANES waves adhered to ethical standards validated by the National Center for Health Statistics, ensuring that written informed consent was acquired from every participant.

Data were obtained from six NHANES cycles conducted spanning from 2007 to 2018. Participants were restricted to adults aged ≥20 years who had completed self-reported questionnaires of PA, SD-related answers, and covariates. Ultimately, this screening process yielded a sample size of 25,732 eligible adult participants for subsequent statistical analyses.

### 2.2. Measurement

#### 2.2.1. Physical Activity

Domain-specific PA was assessed using the Global Physical Activity Questionnaire (GPAQ) [31]. In the present study, PA was classified into three domains based on the context of activity participation: OPA, TPA, and LTPA. OPA refers to activities undertaken during occupational tasks, household responsibilities, and yard work. TPA is defined as walking or cycling for transportation purposes, such as commuting and daily travel. LTPA comprises recreational activities, sports, and exercise performed during discretionary time. Participants reported both the weekly frequency and intensity (vigorous and moderate) of OPA and LTPA. For both OPA and LTPA, total minutes of vigorous-intensity PA were multiplied by two and then added to the moderate-intensity minutes to calculate the final minutes for each domain [20].

The total amount of PA was calculated by summing the minutes accumulated across OPA, TPA, and LTPA. According to the PA Guidelines for Americans (recommending a weekly minimum of 150 moderate minutes, 75–150 vigorous minutes, or an equivalent mixture) [32], we categorized the participants into two groups: (1) participants who adhered to the 2018 PA guidelines (the total PA minutes ≥150 min in a typical week) and (2) participants who did not meet the 2018 PA guidelines (the total PA minutes <150 min in a typical week).

#### 2.2.2. Self-Reported or Doctor-Diagnosed Sleep Disorders

Participants’ sleep-related information was evaluated through the household Computer-Assisted Personal Interviewing, CAPI, system. Based on previously established criteria for identifying SD in NHANES studies [33], participants were classified as having SD if they provided an affirmative response to either of the following questions: “Have you ever told a doctor or other health professional that you have trouble sleeping?” and “Have you ever been told by a doctor or other health professional that you have a sleep disorder?” Subsequently, the participants were categorized into SD and non-SD groups. Those who refused to respond or did not know were treated as missing data.

#### 2.2.3. Covariates Determination

Sociodemographic information, lifestyle behaviors, and clinical characteristics were considered potential covariates in the present study. The selection of covariates was based on prior epidemiological evidence, as these factors have been reported to be associated with both PA behaviors and sleep outcomes [34,35,36]. The included covariates consisted of age, gender, ethnicity, education level, marital status, poverty income ratio, smoking status, BMI, hypertension, diabetes, arthritis, gout, liver condition, cardiovascular disease, pulmonary disease, cancer, and depression. Detailed definitions for all covariates are provided in the Supplementary File.

#### 2.2.4. Statistical Analysis

All statistical analyses were conducted using R software version 4.2.2. Given the complex sampling design of NHANES, sample weights, clustering, and stratification were incorporated into all analyses to obtain nationally representative estimates [37]. Sample weights were adjusted by dividing the 2-year survey weights (WTMEC2YR) by the number of cycles (six). Continuous variables were presented as means ± standard deviations, and categorical variables were expressed as absolute numbers (*n*) and percent values (%). Baseline characteristics were compared between participants with and without SD using the *t*-test for continuous variables and the chi-square test for categorical variables. Survey-weighted multivariable logistic regression models were applied to examine the associations between domain-specific PA and SD. Three models were constructed: a Crude model was adjusted for no covariates; Model 1 was adjusted for age, gender, ethnicity, education level, marital status, poverty income ratio, BMI, and smoking status; Model 2 was further adjusted for clinical characteristics (e.g., hypertension, diabetes, and depression). The restricted cubic spline (RCS) was performed based on the fully adjusted model (Model 2) to investigate the dose–response association between PA and SD. In addition, subgroup analyses were conducted to examine whether associations differed across strata of age, gender, ethnicity, education level, marital status, poverty income ratio, BMI, smoking status, and depression. Statistical significance was set a priori at an alpha level of ≤ 0.05.

## 3. Results

### 3.1. Baseline Characteristics

**Figure 1:**
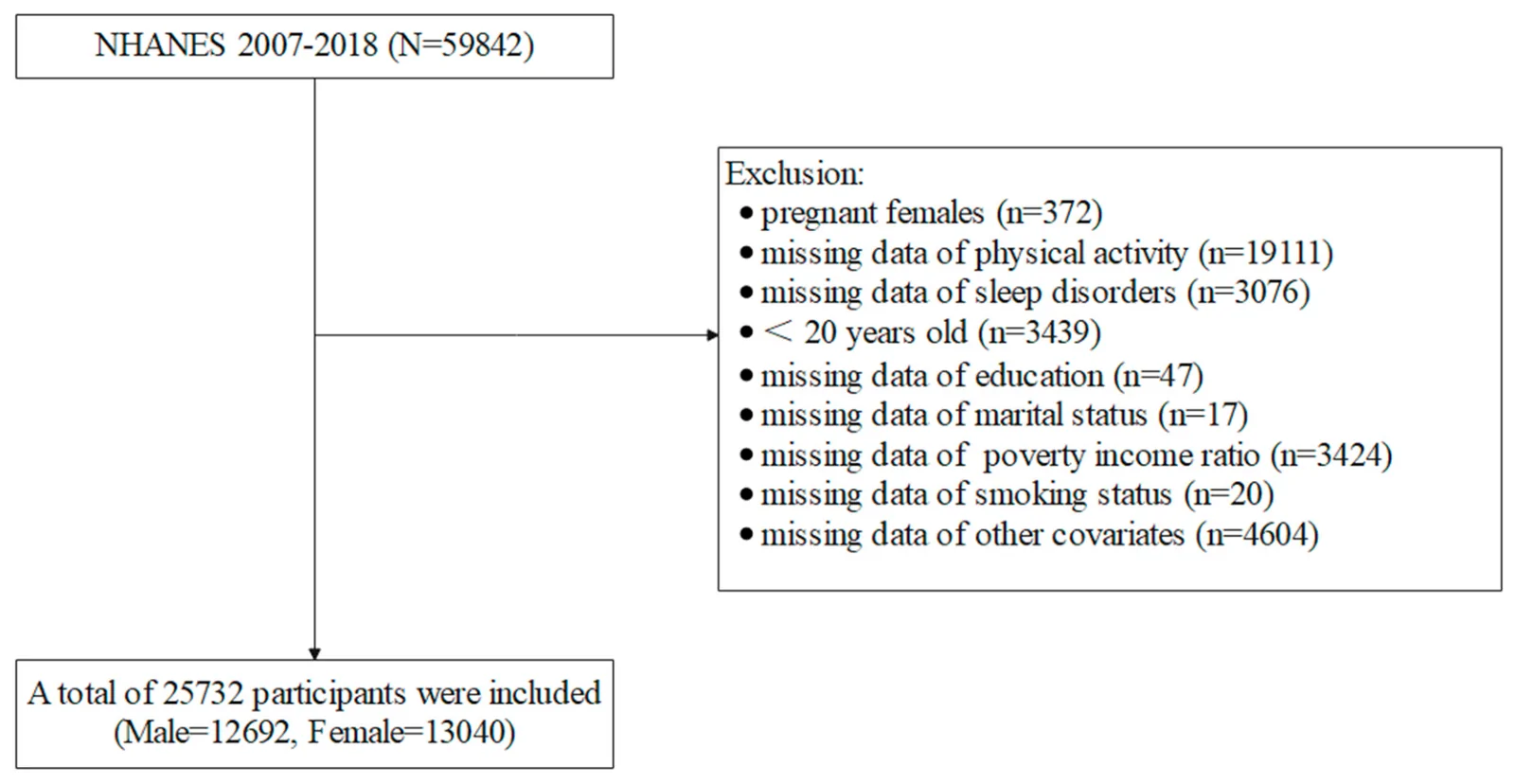
Flowchart of the included participants.

A final sample of 25,732 participants (12,692 male and 13,040 female) from six NHANES cycles (2007–2018) was included in the analyses. The participant selection process is illustrated in Figure 1.

The mean age of participants was 49.72 ± 17.53 years. Among them, 10,641 (41.35%) were classified as young adults, 9010 (35.01%) as middle-aged adults, and 6081 (23.63%) as older adults. Overall, 3571 (13.88%) participants had SD identified through self-reported or physician-diagnosed questionnaire items. According to the 2018 PA guidelines, 15,566 (60.49%) participants achieved the recommended level of total PA (≥150 min/week). Furthermore, 8778 (34.11%), 3480 (13.52%), and 8641 (33.58%) participants achieved the recommended levels of OPA, TPA, and LTPA, respectively. The survey-weighted characteristics of the estimated U.S. population are presented in Table 1, while the unweighted characteristics of the included participants are provided in Supplementary Table S1.

### 3.2. Associations of Total and Domain-Specific Physical Activity with Sleep Disorders

Table 2 summarizes the associations between adherence to the 2018 PA guidelines for different PA domains and SD. In the unadjusted crude model, meeting the recommended level of total PA [odds ratio (OR) 0.75, 95% confidence interval [CI] 0.67–0.85, *p* < 0.001], TPA [odds ratio (OR) 0.67, 95% confidence interval (CI) 0.57–0.78, *p* < 0.001] and LTPA (OR 0.73, 95% CI 0.64–0.83, *p* < 0.001) was associated with lower odds of SD. In contrast, adherence to the recommended level of OPA was not significantly related to SD (OR 1.03, 95% CI 0.92–1.16, *p* = 0.606). After adjusting for demographic characteristics and smoking status (Model 1), participants who met the 2018 PA guidelines for total PA (OR 0.86, 95% CI 0.77–0.96, *p* = 0.012), TPA (OR 0.72, 95% CI 0.62–0.84, *p* < 0.001), and LTPA (OR 0.80, 95% CI 0.71–0.91, *p* < 0.001) had 14%, 28% and 20% lower odds of having SD, respectively. However, participants meeting the recommended level of OPA had 15% higher odds of SD (OR 1.15, 95% CI 1.01–1.30, *p* = 0.031). In the fully adjusted model (Model 2), the associations remained significant only for TPA and OPA. Meeting the recommended level of TPA was associated with lower odds of SD (OR 0.79, 95% CI 0.67–0.93, *p* = 0.006), whereas OPA was associated with increased odds of SD (OR 1.15, 95% CI 1.02–1.30, *p* = 0.023). No significant associations were observed for total PA (OR 1.01, 95% CI 0.89–1.13, *p* = 0.912) or LTPA (OR 0.96, 95% CI 0.85–1.09, *p* = 0.511). 

### 3.3. Dose–Response Relationships Between Domain-Specific Physical Activity and Sleep Disorders

**Figure 2:**
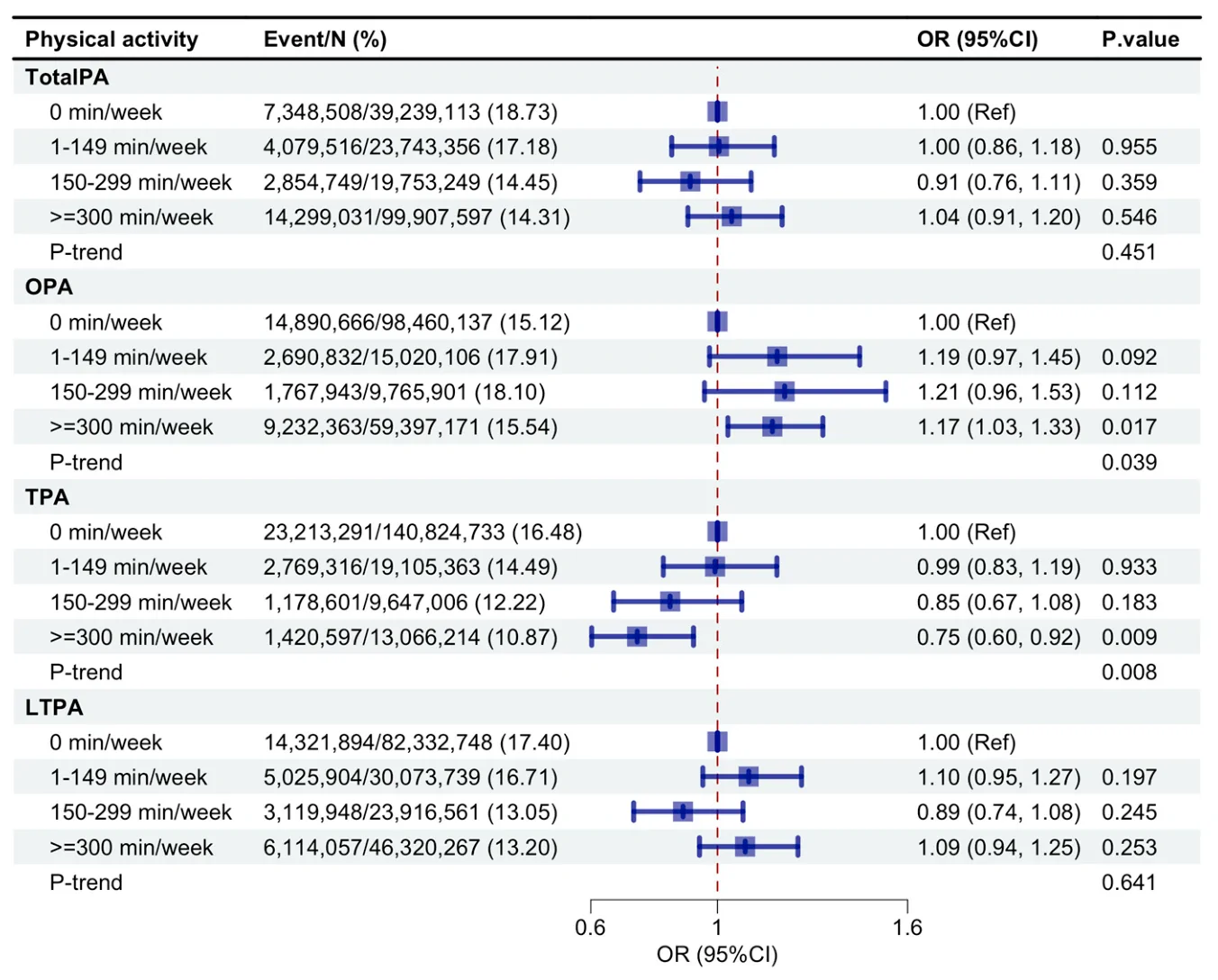
Multivariable OR for SD based on the amount of PA.

To examine the associations between different levels of domain-specific PA and SD, the minutes of PA were stratified into four groups: (1) “inactive”: 0 min/week, (2) “insufficiently active”: 1–149 min/week, (3) “sufficiently active”: 150–299 min/week, and (4) “highly active”: ≥300 min/week (Figure 2). After full adjustment in Model 2, higher OPA levels were associated with increased odds of SD, whereas greater TPA participation was associated with reduced odds of SD. For OPA, relative to inactive participants (0 min/week), those engaging in ≥300 min/week had higher odds of SD (OR 1.17, 95% CI 1.03–1.33, *p* = 0.017). A significant increasing trend was observed across OPA categories (*p*-trend = 0.039). In contrast, a higher level of TPA was associated with lower odds of SD. Compared to inactive participants (0 min/week), those engaging in ≥300 min/week of TPA had lower odds of SD (OR = 0.75, 95% CI: 0.60–0.92, *p* = 0.009), with a significant trend across increasing TPA categories (*p*-trend = 0.008). However, neither total PA nor LTPA categories showed significant associations with SD (*p*-value > 0.05).

**Figure 3:**
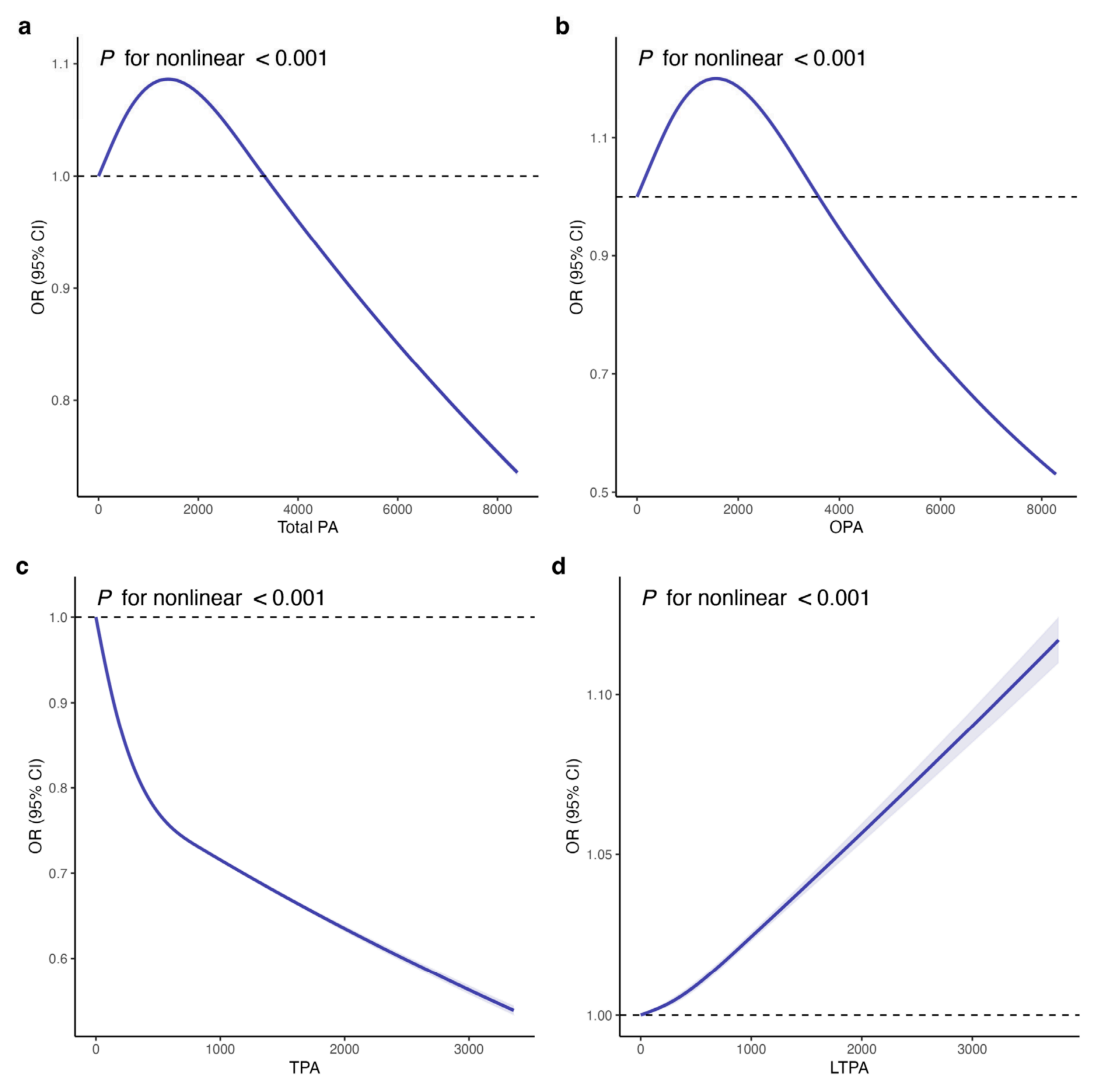
Adjusted restricted cubic spline models showing the associations between different PA domains and the prevalence of SD. (**a**) Association between total PA and SD; (**b**) Association between OPA and SD; (**c**) Association between TPA and SD; (**d**) Association between LTPA and SD. The dashed line represents the reference value (OR = 1.00).

Furthermore, the multivariable-adjusted RCS analysis showed significant nonlinear associations between different PA domains and SD (*p* for non-linear <0.001). Similarly, total PA (Figure 3a) and OPA (Figure 3b) exhibited an inverted U-shaped curve with SD. Specifically, the odds of SD showed an initial increase at lower PA levels, and subsequently decreased as PA levels increased. In addition, TPA demonstrated a nonlinear inverse association with SD (Figure 3c), while LTPA showed a nonlinear positive association (Figure 3d).

### 3.4. Subgroup Analysis

**Figure 4:**
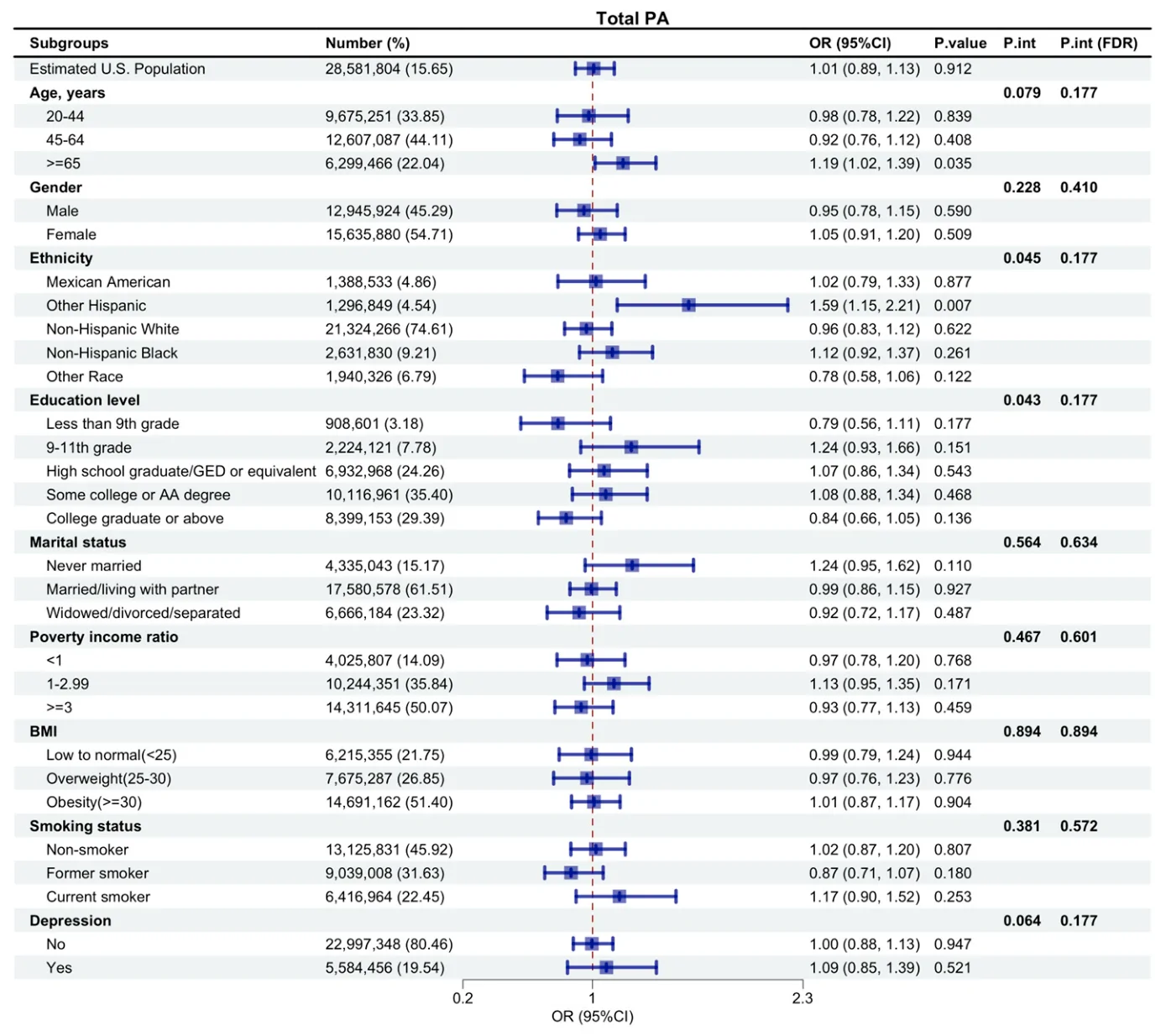
Association of total PA based on meeting PA guidelines with the odds of SD in subgroup analysis. *P*.int (FDR) denotes Benjamini–Hochberg false discovery rate-adjusted *p*-values for interaction tests across subgroup analyses.

Figure 4 displays the subgroup analyses across various strata defined by age, gender, ethnicity, education level, marital status, poverty income ratio, BMI, smoking status, and depression based on Model 2. Except for ethnicity, no significant interactions were observed across most subgroups (*p*-value > 0.05), suggesting that the association between total PA and SD was generally consistent across these subgroups.

**Figure 5:**
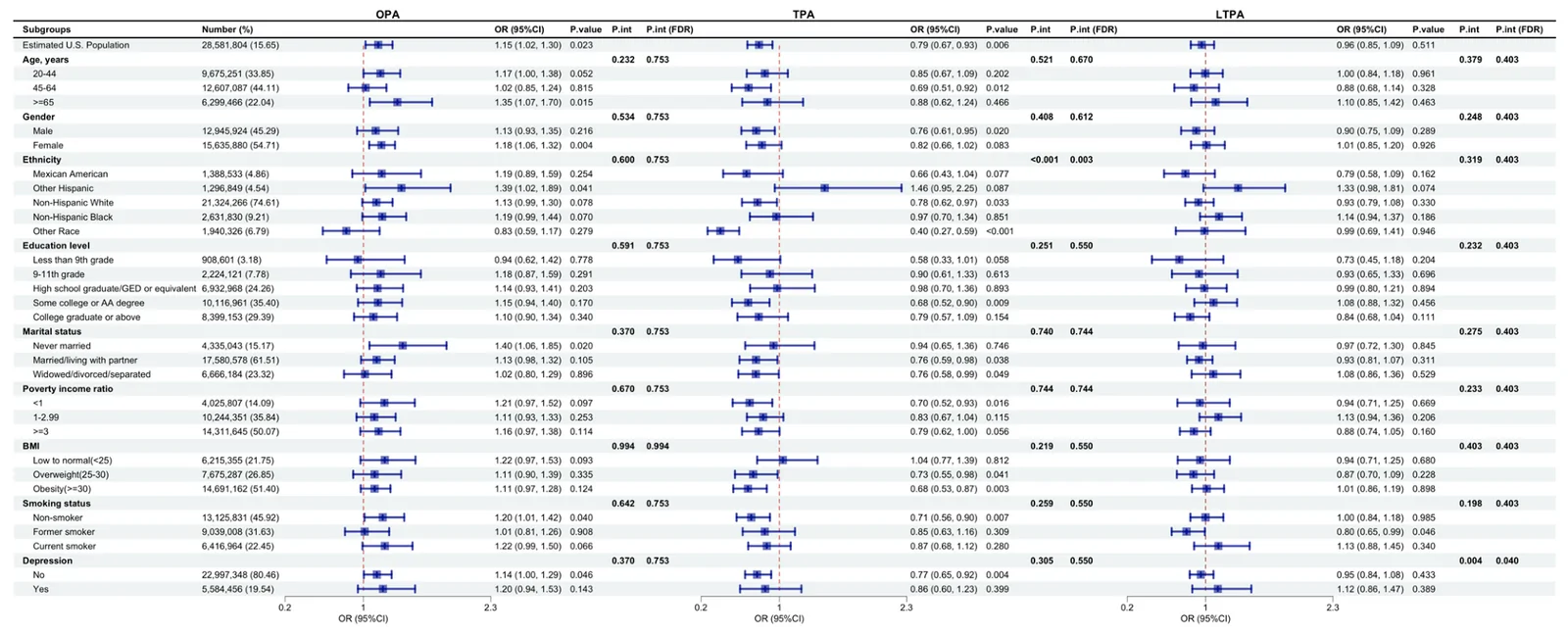
Association of domain-specific PA based on meeting PA guidelines with the risk of SD in subgroup analysis. *P*.int (FDR) denotes Benjamini–Hochberg false discovery rate-adjusted *p*-values for interaction tests across subgroup analyses.

The associations of domain-specific PA with SD across various strata are presented in Figure 5. Significant interactions were identified for TPA by ethnicity (*P*.int < 0.001) and LTPA by depression status (*P*.int = 0.004). Overall, no significant interactions were observed for the remaining subgroup characteristics, suggesting that the associations between domain-specific PA and SD were generally consistent across most subgroups.

## 4. Discussion

While the overall health benefits of PA are widely recognized, the associations between specific PA domains and SD remain insufficiently understood. Using nationally representative data from NHANES 2007–2018, we found that meeting the 2018 PA guidelines for total PA was not associated with lower odds of SD among American adults. For different PA domains, participants meeting the 2018 PA guidelines for OPA had higher odds of SD, while those who met the guidelines for TPA had lower odds of SD. In addition, no significant association was observed between LTPA and SD.

The results of the current study showed that meeting the 2018 PA guidelines for total PA was not associated with reduced odds of SD. Since total PA is the sum of OPA, TPA, and LTPA in our study, the “PA paradox”, which describes the potential contrasting health effects of different PA domains, may partially explain the inability of total PA to significantly improve the SD. In addition, the attenuation of the association between total PA and SD after full adjustment may be partly attributable to over-adjustment. Some clinical covariates included in Model 2 may lie on the pathway between PA and sleep outcomes or represent intermediate factors. Therefore, the fully adjusted model may provide a more conservative estimate. Moreover, the RCS analysis showed a dose–response relationship between total PA and SD (*p* for non-linear <0.001), which implies that the prevalence of SD declined as PA increased after reaching a certain level. Some prior studies have provided some clues about the biological mechanisms underlying the positive effects of PA on SD [38,39,40]. Melatonin is a hormone that regulates crucial physiological processes such as human circadian rhythms and sleep–wake cycles, and its decline of secretion with age can lead to sleep-related problems [38,41,42]. For PA, its protective role on SD is related to the increased secretion of melatonin [39,43,44]. Relevant studies have demonstrated that performing PA could benefit sleep by increasing melatonin release [39,43,45]. More concretely, the mechanism underlying increased melatonin concentrations and improved sleep outcomes is believed to be connected to the effects of melatonin on circadian rhythm changes in core body temperature [44]. It is documented that melatonin reduces heat production and promotes a drop in core body temperature by resulting in vasodilatation, thereby accelerating sleep onset [38,42]. In addition, PA may also modulate the relationships between melatonin, norepinephrine, and adrenaline, potentially allowing for increases in melatonin synthesis and secretion, which might account for PA-induced sleep improvements [40,43].

Additionally, OPA was associated with higher odds of SD after adjusting for sociodemographic information, lifestyle behaviors, and clinical characteristics in the logistic regression analysis, and the RCS model showed a nonlinear relationship between OPA and SD. Part of this may be attributed to high work stress levels and excessive work. A longitudinal study conducted in the United States has shown that higher job strain at baseline was significantly associated with an increase in sleep disturbances across a 9-year follow-up [46]. Furthermore, irregular working hours, such as shift work and night shifts, can seriously disrupt a person’s biological clock, leading to disruption of the sleep cycle, which may cause insomnia, daytime sleepiness, and other problems [47,48]. However, in the current study, an inverse relationship between OPA and SD was observed in the RCS model when the level of OPA approximately reached 2000 min/week. We speculate that the body may develop a certain amount of fatigue when work-related PA reaches a threshold. This fatigue might prompt the body to enter a state of deep relaxation, which makes it easier to fall asleep. Although several potential mechanisms for OPA may explain these associations, these interpretations remain speculative due to the cross-sectional design and lack of information on behavioral context (e.g., occupational stress, work schedules).

The results also revealed that TPA was associated with lower odds of SD, which contradicts some existing studies [27]. For instance, Zheng et al. have found that commuting-related activity is associated with higher risks of insomnia symptoms for both males and females [27]. One possible reason for the inconsistency between existing studies and the current findings may be a result of the differences in the measured PA durations and definitions. In previous studies, PA during the past year was evaluated using other validated questionnaires, which have been previously adopted in several Chinese and Western population studies [49,50]. It is also important to note that commuting-related activity in previous studies referred to PA on the way between home and workplace only, which might be influenced by traffic-related noise and air pollution, thereby contributing to disruptions in sleep quality. However, in our study, TPA was defined as walking or bicycling for traveling from place to place, such as going to school/work or going shopping in a typical week. Therefore, based on the results of the current study, the method by which PA is measured or defined can lead to different interpretations and incongruent results when comparing different studies. The underlying mechanisms of TPA on SD might be interpreted as performing TPA outdoors, which can increase daytime light exposure and help reset the biological clock [51,52,53]. To be specific, Roenneberg et al. developed a questionnaire to evaluate the timing of sleep within the 24 h (sleep phase) quantitatively and indicated that each additional hour spent outdoors per day corresponds to an advance of sleep by almost 30 min [54]. Therefore, the combination of PA and natural daylight might be an additive enhancer of sleep [55].

Interestingly, while logistic regression models utilizing categorical PA groups did not identify significant associations between LTPA and SD, the RCS analysis suggested a positive nonlinear association, which contradicts prior research [56,57]. For example, a study based on the data from the Danish Physical Activity cohort using objective measurements reported that a 10% increase in LTPA was associated with a lower prevalence of non-restorative sleep, which is considered one of the major sleep problems (OR 0.51, 95% CI 0.28–0.92) [56]. Another cohort study of a population-based sample of women verified that a high level of leisure-time PA protected women from future insomnia [57]. However, LTPA did not promote a positive impact on SD, leading the researchers to presume that the time of day the participants exercised may influence their sleep [58]. Along those lines, some studies have speculated that exercising too close to bedtime may lead to hyperarousal, resulting in poor sleep [59]. Furthermore, evening PA could produce circadian phase delays, leading to sleep disruptions [60,61]. In addition, reverse causation cannot be excluded. Individuals with SD may experience fatigue or reduced recovery, which may in turn influence their PA patterns [62,63]. Moreover, measurement error inherent in self-reported PA and SD data and residual confounding may also contribute to these associations.

The significance of the current study lies in its approach to distinguishing different associations between domain-specific PA and SD. Although the observed effect sizes were modest, even small associations may have meaningful public health implications at the population level. Nevertheless, this study has several limitations that should be acknowledged. First, given the cross-sectional correlational study design, a causal relationship between PA and SD cannot be confirmed, and the possibility of reverse causality cannot be excluded. Second, measurement error and recall bias are inherent to the self-reported questionnaires used to assess PA and SD. Third, based on the third edition of the International Classification of Sleep Disorders (ICSD-3), SD are further grouped into seven major categories (e.g., insomnia disorders, sleep-related breathing disorders, and other SD) [64]. However, we could not get the relevant data regarding the specific SD categories, which may introduce outcome heterogeneity. Fourth, the subgroup analyses involved multiple comparisons across several stratified variables, which may increase the likelihood of false-positive findings. Lastly, although we adjusted for possible covariates, we could not avoid the presence of residual confounders such as medication use, obstructive sleep apnea severity, or mental health conditions beyond depression. In addition, some clinical variables included in the fully adjusted model may be associated with both PA and SD and could therefore potentially introduce residual over-adjustment. As a result, the estimates from the fully adjusted model should be interpreted with caution. Therefore, additional studies are needed to collect data on PA domains and SD by objective measurements so more in-depth and precise associations between PA and SD can be better understood.

## 5. Conclusions

The results of this study suggest that TPA was associated with lower odds of SD among U.S. adults based on data from the 2007–2018 NHANES. On the contrary, a higher level of OPA was associated with higher odds of SD. Moreover, there were significant nonlinear dose–response correlations of domain-specific PA with SD. Crucially, these observed associations do not establish causality and must be interpreted strictly within the context of self-reported exposure and outcome measures. Future longitudinal studies and objective PA measurements are needed to refute or confirm the findings of the current study.

## Figures and Tables

**Table 1:** The characteristics of the estimated U.S. population.

	All	Without SD	With SD	*p*-Value
Estimated U.S. population, *n* (%)	182,643,315 (100.00)	154,061,511 (84.35)	28,581,804 (15.65)	
**Age, Mean ± standard deviation**	47.55 ± 16.82	46.84 ± 16.96	51.38 ± 15.50	<0.001
20–44 years	81,976,235 (44.88)	72,300,984 (46.93)	9,675,251 (33.85)	<0.001
45–64 years	67,136,020 (36.76)	54,528,933 (35.39)	12,607,087 (44.11)	
≥65 years	33,531,060 (18.36)	27,231,594 (17.68)	6,299,466 (22.04)	
**Gender, *n* (%)**				<0.001
Male	89,437,572 (48.97)	76,491,649 (49.65)	12,945,924 (45.29)	
Female	93,205,743 (51.03)	77,569,863 (50.35)	15,635,880 (54.71)	
**Ethnicity, *n* (%)**				<0.001
Mexican American	14,482,240 (7.93)	13,093,707 (8.50)	1,388,533 (4.86)	
Other Hispanic	9,766,509 (5.35)	8,469,660 (5.50)	1,296,849 (4.54)	
Non-Hispanic White	125,398,519 (68.66)	104,074,253 (67.55)	21,324,266 (74.61)	
Non-Hispanic Black	19,415,492 (10.63)	16,783,662 (10.89)	2,631,830 (9.21)	
Other race	13,580,555 (7.44)	11,640,228 (7.56)	1,940,326 (6.79)	
**Education level, *n* (%)**				<0.001
Less than 9th grade	8,125,288 (4.45)	7,216,686 (4.68)	908,601 (3.18)	
9–11th grade	18,098,656 (9.91)	15,874,536 (10.30)	2,224,121 (7.78)	
High school graduate/GED or equivalent	41,917,527 (22.95)	34,984,559 (22.71)	6,932,968 (24.26)	
Some college or AA degree	58,119,219 (31.82)	48,002,258 (31.16)	10,116,961 (35.40)	
College graduate or above	56,382,625 (30.87)	47,983,472 (31.15)	8,399,153 (29.39)	
**Marital status, *n* (%)**				<0.001
Never married	33,426,033 (18.30)	29,090,991 (18.88)	4,335,043 (15.17)	
Married/living with partner	115,863,502 (63.44)	98,282,925 (63.79)	17,580,578 (61.51)	
Widowed/divorced/separated	33,353,779 (18.26)	26,687,596 (17.32)	6,666,184 (23.32)	
**Poverty income ratio, *n* (%)**				0.958
<1	25,468,618 (13.94)	21,442,811 (13.92)	4,025,807 (14.09)	
1–2.99	65,185,981 (35.69)	54,941,630 (35.66)	10,244,351 (35.84)	
≥3	91,988,716 (50.37)	77,677,071 (50.42)	14,311,645 (50.07)	
**BMI, *n* (%)**				<0.001
Low to normal	53,373,276 (29.22)	47,157,921 (30.61)	6,215,355 (21.75)	
Overweight	59,771,411 (32.73)	52,096,125 (33.82)	7,675,287 (26.85)	
Obese	69,498,628 (38.05)	54,807,466 (35.58)	14,691,162 (51.40)	
**Smoking status, *n* (%)**				<0.001
Non-smoker	101,565,814 (55.61)	88,439,983 (57.41)	13,125,831 (45.92)	
Former smoker	45,606,321 (24.97)	36,567,312 (23.74)	9,039,008 (31.63)	
Current smoker	35,471,181 (19.42)	29,054,216 (18.86)	6,416,964 (22.45)	
**Depression, *n* (%)**				<0.001
No	168,164,138 (92.07)	145,166,790 (94.23)	22,997,348 (80.46)	
Yes	14,479,178 (7.93)	8,894,722 (5.77)	5,584,456 (19.54)	
Total PA: achieved, *n* (%)	119,660,846 (65.52)	102,507,066 (66.54)	17,153,780 (60.02)	<0.001
OPA: achieved, *n* (%)	69,163,072 (37.87)	58,162,766 (37.75)	11,000,306 (38.49)	0.606
TPA: achieved, *n* (%)	22,713,220 (12.44)	20,114,023 (13.06)	2,599,197 (9.09)	<0.001
LTPA: achieved, *n* (%)	70,236,828 (38.46)	61,002,822 (39.60)	9,234,005 (32.31)	<0.001

Abbreviations: SD, sleep disorders; AA, Associate of Arts; BMI, body mass index; PA, physical activity; OPA, occupation-related PA; TPA, transportation-related PA; LTPA, leisure-time PA.

**Table 2:** Multivariable OR for SD based on meeting PA guidelines.

	Event/*n* (%)	Crude Model		Model 1		Model 2	
Total PA		OR (95% CI)	*p*-Value	OR (95% CI)	*p*-Value	OR (95% CI)	*p*-Value
No	11,428,024/62,982,469 (18.14)	1 (Ref)	1 (Ref)	1 (Ref)	1 (Ref)	1 (Ref)	1 (Ref)
Yes	17,153,780/119,660,846 (14.34)	0.75 (0.67, 0.85)	<0.001	0.86 (0.77, 0.96)	0.012	1.01 (0.89, 1.13)	0.912
OPA							
No	17,581,498/113,480,243 (15.49)	1 (Ref)	1 (Ref)	1 (Ref)	1 (Ref)	1 (Ref)	1 (Ref)
Yes	11,000,306/69,163,072 (15.90)	1.03 (0.92, 1.16)	0.606	1.15 (1.01, 1.30)	0.031	1.15 (1.02, 1.30)	0.023
TPA							
No	25,982,607/159,930,095 (16.25)	1 (Ref)	1 (Ref)	1 (Ref)	1 (Ref)	1 (Ref)	1 (Ref)
Yes	2,599,197/22,713,220 (11.44)	0.67 (0.57, 0.78)	<0.001	0.72 (0.62, 0.84)	<0.001	0.79 (0.67, 0.93)	0.006
LTPA							
No	19,347,799/112,406,487 (17.21)	1 (Ref)	1 (Ref)	1 (Ref)	1 (Ref)	1 (Ref)	1 (Ref)
Yes	9,234,005/70,236,828 (13.15)	0.73 (0.64, 0.83)	<0.001	0.80 (0.71, 0.91)	<0.001	0.96 (0.85, 1.09)	0.511

Note: Model 1 was adjusted for age, gender, ethnicity, education level, marital status, poverty income ratio, BMI, and smoking status; Model 2 was further adjusted for hypertension, diabetes, arthritis, gout, liver condition, cardiovascular disease, pulmonary disease, cancer, and depression in addition to Model 1. Abbreviations: OR, odds ratio; CI, confidence interval.

## Data Availability

The data used in this study are publicly available from the National Health and Nutrition Examination Survey (NHANES) database.

## Supplementary Materials

The following supporting information can be downloaded at: https://www.mdpi.com/article/10.3390/healthcare14162476/s1, Covariates Determination; Table S1: The characteristics of study participants.
