# Supplementary material for: Association of Domain-Specific Physical Activity with Sleep Disorders in U.S. Adults: A Cross-Sectional Study Based on NHANES
Source: Healthcare (Basel). 2026 Aug 10;14(16):2476. doi: 10.3390/healthcare14162476 (PMC13512466; doi:10.3390/healthcare14162476)
Supplement: Supplementary file 1 [file healthcare-14-02476-s001.zip › healthcare-4387856-supplementary.pdf]

Supplementary Material

Covariates Determination

Potential covariates were defined and categorized as follows:

Sociodemographic characteristics included age, gender, ethnicity (Mexican American, Other Hispanic, Non-Hispanic White, Non-Hispanic Black, and Other race), education level [less than 9th grade, 9-11th grade, high school graduate/GED or equivalent, some college or Associate of Arts (AA) degree, and college graduate or above], marital status (never married, married/living with partner, and widowed/divorced/separated), poverty income ratio (<1, 1-2.99, and ≥3).

Lifestyle behaviors included smoking status. Smoking status was classified as non-smoker, former smoker, and current smoker. Individuals who had smoked fewer than 100 cigarettes during their lifetime were defined as non-smokers, whereas those who had smoked at least 100 cigarettes but had quit smoking were classified as former smokers. Individuals who had smoked 100 cigarettes and continued smoking were categorized as current smokers. BMI was calculated as weight divided by height squared (kg/m^2) and categorized as low to normal (≤24.90 kg/m^2), overweight (25-29.90 kg/m^2), and obese (≥30 kg/m^2).

Clinical variables included hypertension, diabetes, arthritis, gout, liver condition, cardiovascular disease (CVD), pulmonary disease, and cancer. Hypertension was based on measured blood pressure values (systolic blood pressure ≥140 mmHg or diastolic blood pressure ≥90 mmHg), or having been diagnosed by a doctor or other health professional. Diabetes was defined based on glycohemoglobin ≥6.50 % or self-reported/physician-diagnosed diabetes. Histories of arthritis, gout, liver condition, CVD (i.e., congestive heart failure, coronary heart disease, angina, heart attack, and stroke), pulmonary disease (i.e., asthma, emphysema, and chronic bronchitis), and cancer were based on whether a healthcare professional had previously informed the participant of such a diagnosis.

Moreover, depression was assessed using the nine-item Patient Health Questionnaire (PHQ-9), which evaluates depressive symptoms experienced during the previous two weeks. The total score ranges from 0 to 27, with each item scored from 0 (not at all) to 3 (nearly every day). A score ≥10 was used to identify participants with possible depressive symptoms.

Table S1. The characteristics of study participants.

	All	Without SD	With SD	*P* Value
Sample, n (%)	25,732 (100.00)	22,161 (86.12)	3,571 (13.88)	
**Age, Mean±standard deviation**	49.72± 17.53	49.17± 17.74	53.12± 15.73	<0.001
20-44 years	10,641 (41.35)	9,545 (43.07)	1,096 (30.69)	<0.001
45-64 years	9,010 (35.01)	7,460 (33.66)	1,550 (43.41)	
≥65 years	6,081 (23.63)	5,156 (23.27)	925 (25.90)	
**Gender, n (%)**				<0.001
Male	12,692 (49.32)	11,088 (50.03)	1,604 (44.92)	
Female	13,040 (50.68)	11,073 (49.97)	1,967 (55.08)	
**Ethnicity, n (%)**				<0.001
Mexican American	3,671 (14.27)	3,302 (14.90)	369 (10.33)	
Other Hispanic	2,540 (9.87)	2,179 (9.83)	361 (10.11)	
Non-Hispanic White	11,140 (43.29)	9,398 (42.41)	1,742 (48.78)	
Non-Hispanic Black	5,426 (21.09)	4,681 (21.12)	745 (20.86)	
Other race	2,955 (11.48)	2,601 (11.74)	354 (9.91)	
**Education level, n (%)**				<0.001
Less than 9th grade	2,316 (9.00)	2,057 (9.28)	259 (7.25)	
9-11th grade	3,487 (13.55)	3,060 (13.81)	427 (11.96)	
High school graduate/GED or equivalent	5,897 (22.92)	5,074 (22.90)	823 (23.05)	
Some college or AA degree	7,799 (30.31)	6,541 (29.52)	1,258 (35.23)	
College graduate or above	6,233 (24.22)	5,429 (24.50)	804 (22.51)	
**Marital status, n (%)**				<0.001
Never married	4,713 (18.32)	4,139 (18.68)	574 (16.07)	
Married/living with partner	15,292 (59.43)	13,303 (60.03)	1,989 (55.70)	
Widowed/divorced/separated	5,727 (22.26)	4,719 (21.29)	1,008 (28.23)	
**Poverty income ratio, n (%)**				0.014
<1	5,369 (20.87)	4,589 (20.71)	780 (21.84)	
1-2.99	10,872 (42.25)	9,321 (42.06)	1,551 (43.43)	
≥3	9,491 (36.88)	8,251 (37.23)	1,240 (34.72)	
**BMI, n (%)**				<0.001
Low to normal	7,301 (28.37)	6,620 (29.87)	681 (19.07)	
Overweight	8,409 (32.68)	7,450 (33.62)	959 (26.86)	
Obese	10,022 (38.95)	8,091 (36.51)	1,931 (54.07)	
**Smoking status, n (%)**				<0.001
Non-smoker	14,219 (55.26)	12,581 (56.77)	1,638 (45.87)	
Former smoker	6,276 (24.39)	5,199 (23.46)	1,077 (30.16)	
Current smoker	5,237 (20.35)	4,381 (19.77)	856 (23.97)	
**Depression, n (%)**				<0.001
No	23,427 (91.04)	20,654 (93.20)	2,773 (77.65)	
Yes	2,305 (8.96)	1,507 (6.80)	798 (22.35)	
Total PA: achieved, n (%)	15,566 (60.49)	13,584 (61.30)	1,982 (55.50)	<0.001
OPA: achieved, n (%)	8,778 (34.11)	7,536 (34.01)	1,242 (34.78)	0.375
TPA: achieved, n (%)	3,480 (13.52)	3,106 (14.02)	374 (10.47)	<0.001
LTPA: achieved, n (%)	8,641 (33.58)	7,650 (34.52)	991 (27.75)	<0.001
